# Polypoid Melanoma: Towards a Dermoscopic Approach

**DOI:** 10.5826/dpc.1104a112

**Published:** 2021-10-01

**Authors:** Camilo Rojas-Erazo, Fernando Valenzuela, Laura Carreño, Francisco González-Coloma

**Affiliations:** 1 Puerto Montt Hospital, Reloncavi Health Service, Puerto Montt, Chile; 2 Department of Dermatology, Faculty of Medicine, University of Chile, Santiago, Chile; 3 Pathology service, Clinical Hospital of the University of Chile, Santiago, Chile

## Case Presentation

**Figure 1:**
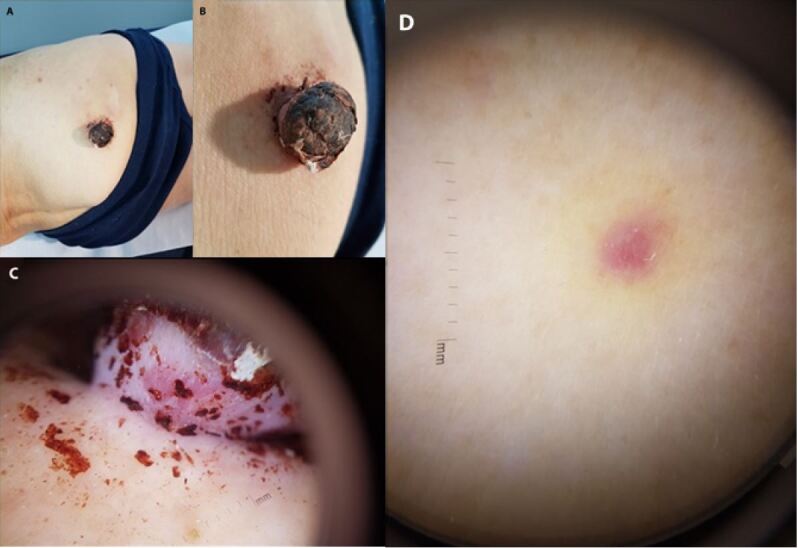
(A) Tumoral lesion on the right gluteus and erythematous papules on the ipsilateral coxal region. (B) Crusty and pigmented surface of the tumor. (C) Polarized dermoscopy (DermLite DL4W, magnification ×10) reveals a multicolored pattern in the peduncle of the tumor and (D) homogeneous reddish center with pigmented periphery of satellite metastasis.

A 75-year-old man presented with a 2-inch exophytic lesion on the right gluteus with 1 year of progressive growth (Figure 1, A and B). Dermoscopy showed an irregular serohematic crusty surface that impaired the observation of atypical polymorphic vessels and a peduncle with multicolored pattern: diffuse red and white background with irregular yellow, brown, and black areas (Figure 1C). Erythematous papules of the ipsilateral coxal region had a central reddish homogeneous pattern with a peripheral pigmented rim at dermoscopy (Figure 1D). Histopathology revealed a polypoid melanoma, Clark V level, Breslow thickness of 14 mm, 12 mitoses/mm^2^, extensive ulceration, and perineural invasion. Coxal papules were satellite metastasis. The dissemination study was negative, yet limited, because of the patient’s death, 1 month later.

## Teaching Point

This rare and aggressive variant of nodular melanoma can be dermoscopically distinguished from clinically similar tumors by the recognition of irregular crusted-fibrinous surfaces, atypical polymorphic vessels, blue-white veils in the exophytic portion, a multicolored pattern, and blue-gray nests at the base [1, 2].
